# DEAttentionDTA: protein–ligand binding affinity prediction based on dynamic embedding and self-attention

**DOI:** 10.1093/bioinformatics/btae319

**Published:** 2024-06-19

**Authors:** Xiying Chen, Jinsha Huang, Tianqiao Shen, Houjin Zhang, Li Xu, Min Yang, Xiaoman Xie, Yunjun Yan, Jinyong Yan

**Affiliations:** Key Lab of Molecular Biophysics of Ministry of Education, College of Life Science and Technology, Huazhong University of Science and Technology, Wuhan 430074, China; Key Lab of Molecular Biophysics of Ministry of Education, College of Life Science and Technology, Huazhong University of Science and Technology, Wuhan 430074, China; Key Lab of Molecular Biophysics of Ministry of Education, College of Life Science and Technology, Huazhong University of Science and Technology, Wuhan 430074, China; Key Lab of Molecular Biophysics of Ministry of Education, College of Life Science and Technology, Huazhong University of Science and Technology, Wuhan 430074, China; Key Lab of Molecular Biophysics of Ministry of Education, College of Life Science and Technology, Huazhong University of Science and Technology, Wuhan 430074, China; Key Lab of Molecular Biophysics of Ministry of Education, College of Life Science and Technology, Huazhong University of Science and Technology, Wuhan 430074, China; Key Lab of Molecular Biophysics of Ministry of Education, College of Life Science and Technology, Huazhong University of Science and Technology, Wuhan 430074, China; Key Lab of Molecular Biophysics of Ministry of Education, College of Life Science and Technology, Huazhong University of Science and Technology, Wuhan 430074, China; Key Lab of Molecular Biophysics of Ministry of Education, College of Life Science and Technology, Huazhong University of Science and Technology, Wuhan 430074, China

## Abstract

**Motivation:**

Predicting protein–ligand binding affinity is crucial in new drug discovery and development. However, most existing models rely on acquiring 3D structures of elusive proteins. Combining amino acid sequences with ligand sequences and better highlighting active sites are also significant challenges.

**Results:**

We propose an innovative neural network model called DEAttentionDTA, based on dynamic word embeddings and a self-attention mechanism, for predicting protein–ligand binding affinity. DEAttentionDTA takes the 1D sequence information of proteins as input, including the global sequence features of amino acids, local features of the active pocket site, and linear representation information of the ligand molecule in the SMILE format. These three linear sequences are fed into a dynamic word-embedding layer based on a 1D convolutional neural network for embedding encoding and are correlated through a self-attention mechanism. The output affinity prediction values are generated using a linear layer. We compared DEAttentionDTA with various mainstream tools and achieved significantly superior results on the same dataset. We then assessed the performance of this model in the p38 protein family.

**Availability and implementation:**

The resource codes are available at https://github.com/whatamazing1/DEAttentionDTA.

## 1 Introduction

Recent research has indicated that predicting drug–target interactions (DTIs) plays a crucial role in virtual drug screening (Lim *et al.* 2019, Luo *et al.* 2021). Furthermore, interactions between proteins and ligands with a high affinity for DTIs play crucial roles in early-stage drug screening (Gilson and Zhou 2007). In medicinal chemistry, the focus is on identifying the key proteins whose biochemical functions can be clearly associated with disease. In fact, a ligand would be considered effective if it binds with high affinity at the binding site and has an effective therapeutic effect (Dhakal *et al.* 2021). In the context of preliminary virtual drug screening, predicting low-affinity small-molecule drugs is crucial for their exclusion. Intermolecular interactions between proteins and ligands occur at specific positions in proteins, known as ligand binding sites. Binding sites, also known as binding pockets, are usually depressions on the surface of proteins (Krivák and Hoksza 2015). For example, the p38 MAP kinase protein possesses an allosteric binding site, allowing it to modulate protein function through direct compound interactions. This binding event triggers substantial conformational changes, resulting in kinase activity inhibition, offering potential therapeutic strategies for managing inflammation (Pargellis *et al.* 2002). The affinity between a protein and a ligand represents the binding strength between them and is typically expressed by inhibition constants (e.g. Ki), dissociation constants (e.g. Kd), and half-maximal inhibitory concentrations (e.g. IC50) (Rose *et al.* 2017). Currently, experimental methods for determining affinity include surface plasmon resonance (Olaru *et al.* 2015), enzyme-linked immunosorbent assay (Lindstrom and Wager 1978), and isothermal titration calorimetry (Transtrum *et al.* 2015). Although these experimental methods are accurate, they are time-consuming and costly. Therefore, computational prediction methods (Dhakal *et al.* 2021) are gaining increasing attention because of their efficiency (Abbasi *et al.* 2020).

Computational prediction methods can be broadly categorized into traditional machine learning and the emerging field of deep learning. Traditional machine learning methods, such as support vector machines, are employed for binary predictions of DTIs (Cao *et al.* 2012), which involve determining whether there is an interaction between proteins and ligands. They require setting a threshold, where interactions above the threshold are considered positive and those below are considered negative. Research indicates that deep-learning-based models outperform those based on traditional machine learning in terms of prediction accuracy (Karimi *et al.* 2019, Öztürk *et al.* 2019, Rifaioglu *et al.* 2019). It is because protein sequences contain many complex information, making it challenging for manual feature extraction to be effective. With the emergence of an increasing number of publicly available protein datasets, models based on deep learning for the large-scale prediction of drug–target affinity (DTA) have been continuously emerging (Davis *et al.* 2011, Tang *et al.* 2014, Liu *et al.* 2017).

Pafnucy (Stepniewska-Dziubinska *et al.* 2017) utilizes the 3D information of proteins by inputting the coordinates of proteins and small molecules into a 3D convolutional neural network (3DCNN). The final step involves regression fitting through three fully connected layers. DLSSAffinity (Wang *et al.* 2022) extends this approach by separating the protein and ligands. The 3D structure of the protein is input into a 3DCNN, while the linear sequences of amino acids and small molecules are input into a regular convolutional neural network (CNN). The outputs of these components are then concatenated, and regression fitting is performed through three fully connected layers. DeepDTAF (Wang *et al.* 2021) utilizes manually pre-defined features of amino acids as the input. These features include physicochemical properties and secondary structure characteristics, among others. Linear sequences and small ligand molecules are concatenated using multiple CNNs, with fully connected layers serving as the final output. Despite the favourable results, there is no definitive conclusion regarding the effectiveness of manually extracted features. GraphDTA (Nguyen *et al.* 2021) and GraphscoreDTA (Wang *et al.* 2023) directly input the 3D structures of protein molecules and ligands into neural networks using a voxel-wise approach. Prediction is then performed using graph neural networks. The advantage lies in utilizing the complete 3D information of both proteins and ligands, allowing the network to fit to the maximum extent; however, it faces limitations when dealing with proteins whose 3D structures are unknown. With continuous breakthroughs in natural language processing technologies in recent years, deep-learning models that rely on linear sequence inputs rather than 3D structures have emerged. The DTI-RCNN (Zheng *et al.* 2018) employs a combination of long short-term memory network and CNN methods for affinity prediction.

In this study, we developed a model named DEAttentionDTA that predicts protein–ligand affinity using three inputs: a 1D amino acid sequence, a 1D active pocket sequence, and a 1D small-molecule sequence. Diverging from various existing tools, DEAttentionDTA draws inspiration from the neural network ELMo (Peters *et al.* 2018). It departs from static word-embedding methods, opting for dynamic input word embeddings. The vector for each amino acid is determined not only by itself but also by the surrounding vectors, allowing the model to capture both long- and short-range information. Our findings collectively demonstrated the effectiveness of DEAttentionDTA for protein–ligand affinity prediction.

## 2 Materials and methods

### 2.1 Datasets

In this study, we utilized the PDBbind database (Liu *et al.* 2017), a dataset comprising protein–ligand binding affinity data, with the structure files and affinities of protein–ligand complexes obtained from the publicly available Protein Data Bank (Burley *et al.* 2019). Affinities are represented as −logKi, −logKd, or −logIC50. We utilized the 2020 version of the PDBbind database, which comprises 19 420 protein–ligand complexes. Additionally, two high-quality datasets, CASF2016 (Su *et al.* 2019) and CASF2013 (Li *et al.* 2018), comprising 285 196 protein–ligand complexes, were used for validation. Each protein–ligand dataset included protein pdb format files, active pocket pdb format files, and small-molecule sdf format files. The sdf format files for the small molecules were converted to smi format files using OpenBabel (O'Boyle *et al.* 2011). Given that the 3D structures of most proteins are currently unknown, all input sequences used in this model were linear sequences, with only 1D data used as the input for model construction.

In terms of dataset partitioning, given the utilization of a self-attention network structure, *k-*fold cross-validation was employed for 19 420 data pairs. The hyperparameter *k* was set to 10, dividing the dataset into 10-folds. In each iteration, 9-folds were used to train the model, and the remaining fold served as the validation set to assess the model performance. The structural configuration of each model was identical. Consequently, we obtained 10 models using this process. For testing, data from the core2016 test set were fed into these 10 models, resulting in 10 distinct predicted affinity outcomes. The final test result was derived by averaging the predictions of all the models.

**Figure 1.**
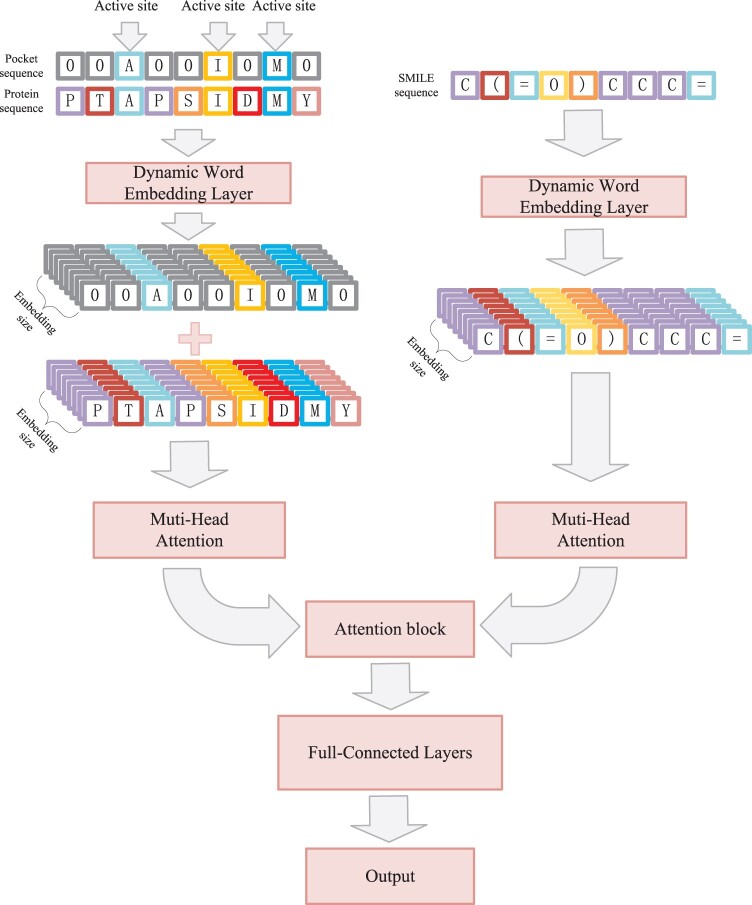
Architecture of the DEAttentionDTA. Three main sequences, including the protein, pocket, and ligand SMILES sequences, were input into the network and fed into a dynamic word-embedding layer. The embedding outputs were fed into self-attention layers. Finally, proteins and ligands interacted through attention blocks, followed by affinity regression prediction through three fully connected layers.

For data preprocessing, to address the variable lengths of amino acids and SMILE sequences, we analysed the lengths of amino acids and SMILE sequences in the training dataset. As shown in the Supplementary Material (Figure 1), the maximum protein sequence length was 4720, and the longest SMILE sequence length was 540. Consequently, we truncated the protein sequences to a fixed length of 1024 and the SMILE sequences to a fixed length of 256. This fixed length range covered 93.3% and 96.7% of the protein and SMILE sequences, respectively. Sequences that exceeded a fixed length were truncated to the specified length, whereas sequences shorter than the fixed length were padded with zeros to reach the fixed length.

### 2.2 Input representation

Owing to the requirement for numerical inputs in neural networks rather than characters, we employed a label-encoding approach to encode the protein and ligand SMILE sequences. Each distinct protein sequence and ligand SMILE sequence was transformed into a unique array using two pre-defined dictionaries. In the protein and SMILE dictionaries, index 0 corresponded to padding. For the sorting of dictionaries, we retrieved all the training set data and constructed a protein dictionary and a ligand dictionary according to the order of word frequency. The higher the word frequency, the higher the ranking in the dictionary, and the first place in the dictionary was ‘<MASK>’ to fill 0. The sorted dictionary can make the gradient calculation more efficient. Because the gradient update frequency of common words is high, the gradient calculation for these words will be more frequent and concentrated in the back propagation, which helps to converge faster. The protein dictionary, including padding, comprised 21 elements, whereas the SMILE dictionary, including padding, comprised 53 elements. All pre-defined dictionaries of protein and ligand sequences are listed in the Supplementary Material. For a protein sequence, given a pre-defined dictionary such as {‘A’: 1, ‘D’: 3, ‘P’: 13, ‘S’: 16, ‘T’: 17}, the sequence ‘PTAPSD’ would be transformed as follows:

[P T A P S D]=[13 17 1 13 16 3].

For the ligand's SMILE sequence, given the pre-defined dictionary {‘C’: 1, ‘O’: 5, ‘(’: 2, ‘)’: 3, ‘=’: 10}, the sequence ‘C(=O)CCC’ would be transformed as follows:

[C (=O) C C C]=[1 2 10 5 3 1 1 1].

### 2.3 Model construction

#### 2.3.1 Dynamic word-embedding layer

In this study, we applied a dynamic word-embedding network model based on a self-attention mechanism to predict the protein–ligand binding affinity. The overall network structure is illustrated in Figure 1.

**Figure 2.**
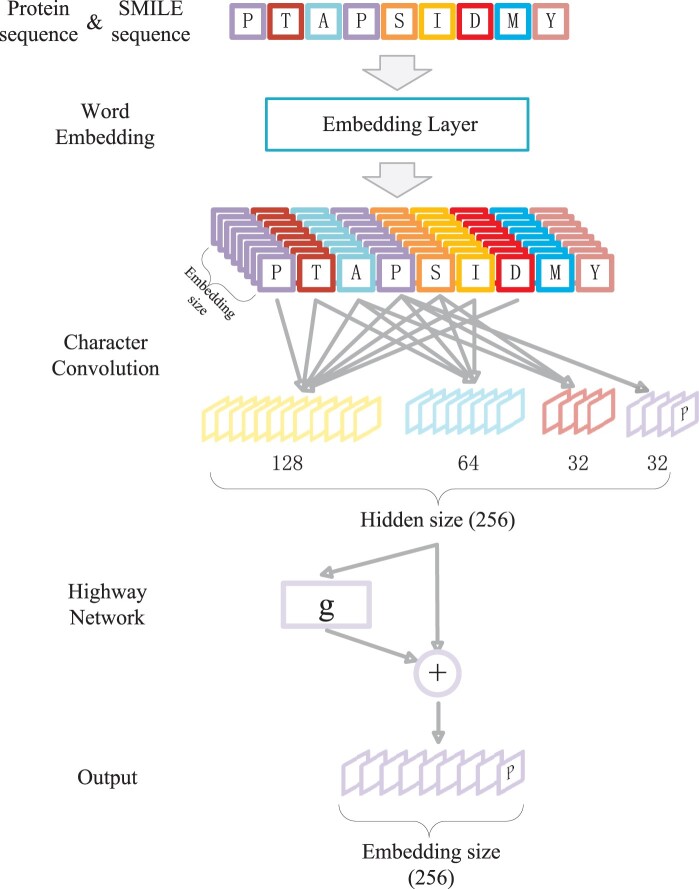
Architecture of dynamic word-embedding layer. The protein sequences inputted into the network are initially represented by word-embedding through embedding layers, followed by feature extraction through 1DCNN, and finally gated out through the highway network.

The protein and ligand SMILE sequences, encoded into lists of positive numbers, were inputted into the dynamic word-embedding layer. A specific model of the dynamic word-embedding layer is illustrated in Figure 2. The 1D sequences of the proteins and ligand SMILE were input into the embedding layer and mapped to $R^{L\times E}$, where *L* is the fixed length of the sequence and *E* is the embedding size. The dimensions *L* and *E* were then swapped, transforming the dimensional space into $R^{E\times L}$. A 1D convolution was applied in the *L* dimension with kernel sizes of 1, 3, 5, and 7, corresponding to 32, 32, 64, and 128 convolutional kernels, respectively.

The first 32 dimensions of the four convolutional outputs contain information about the target amino acid itself, the second 32 dimensions contain information about the three neighbouring amino acids of the target amino acid, the third 64 dimensions contain information about the five neighbouring amino acids of the target amino acid, and the last 128 dimensions contain information about the seven neighbouring amino acids of the target amino acid. These outputs were then concatenated into a 256D feature vector, transforming the dimensional space into $R^{H\times L}$, where *H* is the hidden size with a specific value of 256. This operation results in the final concatenated output containing information not only on the target amino acid itself but also about the surrounding amino acids. Each position's amino acid obtains a unique word-embedding representation, enabling the neural network to better comprehend the information surrounding the amino acids. By utilizing multiple convolutional kernel sizes, the model captured context information from different granularities of the sequence and obtained the corresponding hidden layer vector representations. Subsequently, the model further transformed the output of the CNN using a highway neural network to obtain the final dynamic word vectors. A highway neural network is a gated neural network that directly establishes a shortcut between input and output to avoid exploding or vanishing gradients. This enables the gradient to be directly back-propagated to the input layer. The specific calculation formula for a single-layer highway neural network is as follows:

(1) $$x_{t}=g⊙f_{t}+\left(1-g\right)⊙ReLU\left(Wf_{t}+b\right)$$

In the above formula, $f_{t}$ represents the input and g represents the gating vector. $f_{t}$ was obtained by applying a linear transformation followed by a sigmoid activation function:

(2) g=σWgft+bg

After passing through the highway neural network, the output space was $R^{E\times L}$. Finally, another transposition of dimensions occurred, swapping *L* and *E*, resulting in the final word vectors in $R^{L\times E}$.

In version 1, we introduced local sequence information by adding an active pocket sequence (which had the same length as the protein sequence, with positions other than the active site filled with zeros) to the protein sequence after the dynamic word-embedding layer. The addition was performed position-wise to ensure that the network paid more attention to the active site than that to the ordinary positions. In version 2, only the protein sequence was used as the input. The SMILE sequence was processed through the dynamic word-embedding layer in the same manner in both versions.

#### 2.3.2 Multi-head attention layer

Each amino acid contains multiple semantic features such as acidity, alkalinity, polarity, non-polarity, and residue size. In contrast to a regular attention mechanism, a multi-head attention mechanism was employed to fully extract various types of semantic information/features. The feature dimensions were divided into *k* parts, and the attention mechanism was applied separately to each part. Finally, the results were concatenated, which is the principle behind the multi-head attention mechanism.

The first step involved setting the number of heads to *k*. The output from the previous step with an embedding size was divided into *k* equal parts, resulting in *k* different $X_{i}$. Each $X_{i}$ was then mapped to three matrices $Q_{i}K_{i}V_{i}$ using three linear layers without bias:

(3) Qi=XiWiqi

(4) Ki=XiWiki

(5) Vi=XiWivi

Next, the three matrices $Q_{i}K_{i}V_{i}$ were input into the attention calculation to obtain the final ${\mathrm{head}}_{i}$. During the attention operation, the matrix product of *Q* and the transpose of *K* were divided by $\sqrt{d_{k}}$, where $\sqrt{d_{k}}$ acted to normalize the mean and variance of the entire output data, making them approach a Gaussian distribution. This reduced data fluctuations, similar to normalization, and prevented the input to the softmax activation function from becoming excessively large, which could lead to vanishing gradients

(6) headi=AttentionQi,Ki,Vi=SoftmaxQi×KiTdk×Vi

Finally, the *k* heads were concatenated to form a multi-dimensional feature, which was the output

(7) Muti-HeadQ,K,V=Concathead1,head2,…,headhWO

#### 2.3.3 Integrating attention block and fully connected output block

In this module, the final attention result $\alpha \in R^{L_{P}\times L_{S}}$ was obtained by performing a dot operation on the protein sequence $P\in R^{L_{P}\times E}$ and the SMILE sequence ${S\in R}^{L_{S}\times E}$ through the self-attention mechanism layer:

(8) α=P×ST

An Adaptive-AveragePooling layer was applied to *α*, resulting in 1D output $\beta \;\in R^{L_{P}}$

(9) β=AdaptiveAvgPool1dα

Finally, $\beta \;\in R^{L_{P}}$ was input into three fully connected layers (FC), with the number of neurons in each layer being 256, 64, and 1, respectively. Dropout layers, which temporarily deactivated certain neurons during forward propagation with probability *p*, were incorporated into each fully connected layer to enhance the generalization of the model by preventing excessive reliance on specific local features.

After each dropout layer, an activation function was applied to better capture the nonlinear relationships. In terms of the choice of activation function, we opted for its variant, PReLU, over a regular ReLU function. This decision was made because the standard ReLU function outputs 0 for values less than 0, which potentially causes neuronal death

(10) fx=max⁡0,x+α min⁡0,x 

where *α* is a learnable parameter.

### 2.4 Training settings

To optimize DEAttentionDTA, we chose AdamW (Loshchilov and Hutter 2017) as the network optimizer. In traditional SGD, regularization terms (L2 and L1 regularizations) are typically introduced to increase the generalization ability of the model. With the introduction of the L2 regularization term, the result of the gradient computation for the regularization term was added when computing the gradient. Therefore, if the parameters were relatively large, the corresponding gradients were also large. In Adam’s calculation, the numerator is divided by the cumulative square of the gradient, making the subtraction term relatively small. This results in Adam being unable to penalize excessively large weights.

AdamW correctly introduces weight decay. First, let $\nabla f_{t}(x_{t-1})$ be the gradient at time *t* and let $\beta_{1}$ and $\beta_{2}$ be the moving rates for the first and second moments of the gradient, respectively. Then we have:

(11) gt=∇ftxt−1+wxt−1

(12) vt=β1vt−1+1-β1gt

(13) st=β2st−1+1-β2gt2

(14) v^t=vt1-β1t

(15) s^t=st1-β2t

(16) xt=xt−1-ηtαv^ts^t+ε+λxt−1

Due to the much lower moving speed of the second moment of the gradient compared to the first moment, $\beta_{1}$ and $\beta_{2}$ were set to 0.9 and 0.999, respectively. The learning rate *α* was set to 0.0001, and $v_{0}$ and $s_{0}$ were initialized to 0. $\eta_{t}$ is a trainable parameter that was updated continuously during training.

In terms of training epochs, to prevent the model from overfitting or underfitting, we adopted an early-stop training strategy. The maximum number of epochs was set to a hyperparameter of 50. The training was stopped in two cases: when the training epoch reached 50 or when the training set loss did not decrease continuously for three consecutive epochs and was less than the validation set loss.

We used an embedding layer to map the amino acids to a 128D vector. In the multi-head attention layer, the number of heads was set to 8, the dimensions of the d_model were changed to 16, and the number of layers was set to 6. The neurons in the final fully connected layers were configured as follows: 1024, 256, 64, and 1 for each layer.

All algorithmic strategies in DEAttentionDTA were based on PyTorch (Paszke *et al.* 2019), and we conducted model training using a Linux server equipped with 52 CPUs and an Nvidia GeForce RTX 3090 GPU with 24 GB of VRAM.

### 2.5 Evaluation metrics

To evaluate the protein–ligand affinity prediction results, we used various indicators to evaluate the performance of the DEAttentionDTA model, including the root mean square error (RMSE), mean absolute error (MAE), consistency index (CI), Pearson correlation coefficient (*R*), and standard deviation (SD). The specific formulas are as follows:

(17) RMSEy,y^=1N∑i=1Nyi-y^i2

(18) MAEy,y^=1N∑i=1Nyi-y^i

(19) CI=1Z∑yi > yjfy^i,y^j

(20) fy^i,y^j=1, if y^i>y^j0.5, if y^i=y^j0, if y^i<y^j

(21) Ry,y^=∑i=1nyi-yi¯y^i-y^i¯∑i=1nyi-yi¯2∑i=1ny^i-y^i¯2

(22) SDy,y^=1N−1∑i=1nyi-ay^i+b

The CI was calculated as follows: The CI refers to the proportion of all pairs of samples where the predicted results are consistent with the actual results. It estimates the probability of the predicted results being consistent with the observed results. For any two samples *i* and *j* selected from all samples, if $y_{i}\;$was >${\;y}_{j}$ and if ${\hat{y}}_{i}\;\mathrm{was}>\;{\hat{y}}_{j}$, then this sample pair was considered a positive sample pair. If ${\hat{y}}_{i}\;\mathrm{was}\;=\;{\hat{y}}_{j}$, it was considered a semi-positive sample pair. Otherwise, it was considered a negative sample pair. Finally, the proportion of positive sample pairs among all sample pairs was calculated to obtain the CI.

A good model should have lower RMSE, MAE, and SD values, and higher CI and *R* values.

## 3 Results

### 3.1 Comparison with competing methods

As the structures of only 100 000 proteins are currently resolved, and there are billions of proteins and peptides with known sequences but unknown structures, as well as unknown active pocket sequences and positions (Steinegger *et al.* 2019, Mitchell *et al.* 2020), we designed two versions of the model. Version one required the protein sequence, active pocket sequence, positional information, and a small-molecule SMILE sequence. In this version, we incorporated the pocket sequence information into the protein sequence without rigid concatenation. Instead, we added them position-wise, ensuring that the model paid specific attention to the location of the active pocket in the sequence rather than averaging over each amino acid. The integration of local and global information sequences along with the SMILE sequence involved self-attention layers, enabling the model to model the dependencies between any two positions in the sequence without distance limitations. This allowed the model to capture long-range dependencies and better understand the contextual information in the sequence data. Version two relied solely on the protein sequence and small-molecule SMILE sequence as inputs. The SMILE and protein sequences interacted through attention block layers, followed by several fully connected layers for the regression task output. In addition, we tested our models on a public dataset and compared the results with those obtained using various popular tools.

**Figure 3.**
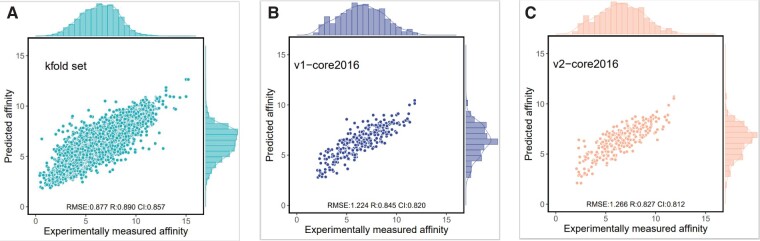
Distributions of the predicted affinities in the *k*-fold set for DEAttentionDTA (A), Core 2016 set for DEAttentionDTA (B), and Core 2016 set for DEAttentionDTA-v2 (C).

**Figure 4.**
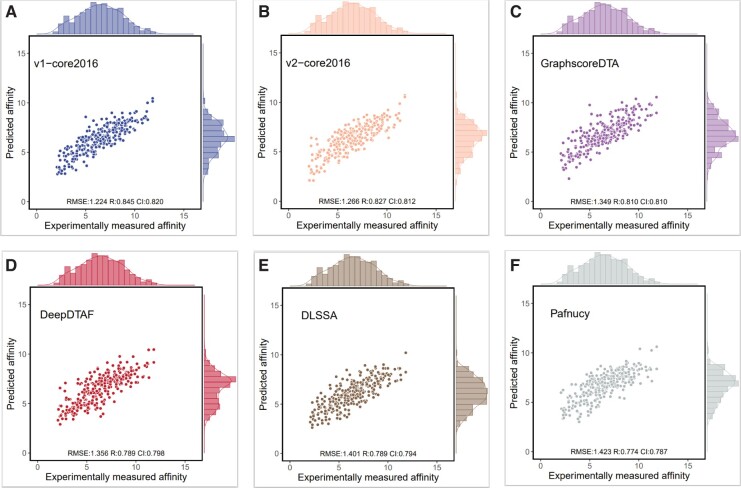
The performance of DEAttentionDTA (A), DEAttentionDTA-v2 (B), GraphscoreDTA (C), DeepDTAF (D), DLSSAffinity (E), and Pafnucy (F) in the core 2016 test set for the prediction of binding affinity.

To evaluate the performance of DEAttentionDTA (both version one and version two)in predicting protein–ligand affinity, we compared our model with several existing tools on the CASF-2016 (Su *et al.* 2019) and CASF-2013 datasets (Li *et al.* 2018). The models included in the comparison were Pafnucy (Stepniewska-Dziubinska *et al.* 2017), DLSSAffinity (Wang *et al.* 2022), DeepDTAF (Wang *et al.* 2021), and GraphscoreDTA (Wang *et al.* 2023) models. The performance of DEAttentionDTA on the PDBbind dataset is shown in Table 1 and Figure 3. The results of comparison with other tools on core 2016 are presented in Table 2 and Figure 4. Since core 2013 does not provide pocket files, the v2 version was used for comparison with the other tools in core 2013, and the results are presented in Table 3.

**Table 1.** Performance of DEAttentionDTA (v1 and v2).

Dataset	RMSE↓	MAE↓	SD↓	CI↑	*R*↑
K_fold	0.877	0.661	0.843	0.857	0.890
V1-Core2016	1.224	1.003	1.166	0.820	0.845
V2-Core2016	1.266	1.014	1.222	0.812	0.827

**Table 2.** Performance of DEAttentionDTA and other competing methods in the core 2016 test set.

Methods	RMSE↓	MAE↓	SD↓	CI↑	*R*↑
DEAttentionDTA	1.224	1.003	1.166	0.820	0.845
DEAttentionDTA-v2	1.266	1.014	1.222	0.812	0.827
GraphscoreDTA	1.349	1.053	1.281	0.810	0.810
DeepDTAF	1.356	1.075	1.337	0.798	0.789
DLSSAffinity	1.401	1.134	1.336	0.794	0.789
Pafnucy	1.423	1.135	1.378	0.787	0.774

**Table 3.** Performance of DEAttentionDTA-v2 and other competing methods in the core 2013 test set.

Methods	RMSE↓	MAE↓	SD↓	CI↑	*R*↑
DEAttentionDTA-v2	1.359	1.102	1.292	0.810	0.819
GraphscoreDTA	1.542	1.236	1.479	0.780	0.758
DeepDTAF	1.791	1.514	1.780	0.732	0.758
DLSSAffinity	1.596	1.304	1.584	0.746	0.732
Pafnucy	1.623	1.318	1.613	0.753	0.697

As presented in Table 2, we observed that DEAttentionDTA achieved the best performance in each evaluation metric compared with GraphscoreDTA, DeepDTAF, DLSSAffinity, and Pafnucy. Regarding the RMSE metric, DEAttentionDTA outperformed GraphscoreDTA, DeepDTAF, DLSSAffinity, and Pafnucy by 9.27%, 9.73%, 12.63%, and 13.98%, respectively. For the MAE metric, DEAttentionDTA outperformed the aforementioned tools by 4.75%, 6.70%, 11.55%, and 11.63%, respectively. For the SD metric, DEAttentionDTA demonstrated enhancements of 8.98%, 12.79%, 12.72%, and 15.38% compared to GraphscoreDTA, DeepDTAF, DLSSAffinity, and Pafnucy, respectively. The CI metric improvements were 1.23%, 2.75%, 3.27%, and 4.19%, respectively. Moreover, the R metric improvements were 4.32%, 6.91%, 6.91%, and 9.17%, respectively.

The most significant improvement was observed in the SD metric, where DEAttentionDTA achieved a 0.093 improvement over the second-best result. The smallest improvement was observed in the CI metric, with a 0.010 improvement over the second-best result. The v2 version, compared with the regular version, showed performance losses of only 3.43%, 1.09%, 6.47%, 0.96%, and 2.13% in the RMSE, MAE, SD, CI, and *R* metrics, respectively. This indicates that the v2 version, which did not require pocket information, remained flexible while maintaining good performance.

### 3.2 The effects of different components or hyperparameters of the model

In this study, we investigated the impact of several modules in the DEAttentionDTA model on the final results. We conducted experiments in which we removed the dynamic word-embedding layer and replaced it with static word embedding for Model 1. For Model 2, we removed the self-attention layer. For Model 3, we removed the attention block and concatenated the protein sequences directly to small-molecule sequences. For Model 4, we added positional encoding to the input of the multi-head attention mechanism. Table 4 presents these results.

**Table 4.** Performance of DEAttentionDTA without dynamic word embedding, without self-attention, without attention block, and additional positional encoding.

Methods	RMSE↓	MAE↓	SD↓	CI↑	*R*↑
DEAttentionDTA	1.224	1.003	1.166	0.820	0.845
Model I	1.389	1.096	1.273	0.785	0.754
Model II	1.482	1.169	1.297	0.766	0.724
Model III	1.683	1.403	1.479	0.701	0.704
Model IV	1.357	1.184	1.298	0.759	0.784

Next, we explored the impact of the number of different heads on the prediction results of the model. We selected 1, 2, 4, 8, 16 as the number of heads. When the number of head is 1, the multi-head attention degenerates into ordinary self-attention. Table 5 presents these results.

**Table 5.** Performance of DEAttentionDTA with different number of heads.

Number of heads	RMSE↓	MAE↓	SD↓	CI↑	*R*↑
1	1.282	1.034	1.244	0.811	0.820
2	1.267	1.012	1.214	0.815	0.829
4	1.267	0.998	1.236	0.812	0.823
8	1.224	1.003	1.166	0.820	0.845
16	1.243	0.981	1.203	0.812	0.833
32	1.274	1.024	1.233	0.811	0.824

From the results in Table 4, it can be observed that the dynamic word-embedding layer has a positive effect on the model because its word vector will be fine-tuned or updated according to the gradient of the model’s loss function, which can better reflect the location of amino acids and the surrounding amino acid information. Each attention head of the multi-head attention mechanism can learn various attention weights to capture the relationship between different parts of the input sequence. As the module for interaction between protein sequences and small-molecule sequences, the attention block has the greatest impact on the prediction results, achieving cross-features. This helps capture the correlation and interaction information in the input data, providing a richer and more complex feature representation. Directly concatenating two matrices without performing feature cross-operation makes the interaction between different features less evident, limiting the model's ability to learn complex relationships in the data. Finally, adding position encoding to the model actually decreases the prediction performance. This is because the dynamic word-embedding layer has already extracted the positional information of the amino acid sequence. Introducing position encoding at this point would introduce redundant information, making the position encoding added in the transformer unsuitable for this task.

From the results in Table 5, it can be observed that when the number of heads is 8, the RMSE, CI, SD, and *R* metrics are the best. When the number of heads is too large, the model may overfit to the noise and details in the training data, losing its ability to generalize to the overall characteristics of the data, ultimately leading to overfitting. Conversely, when the number of heads is too small, the model captures fewer types of attention weights, potentially resulting in a lack of attention to different parts of the input sequence when understanding the data.

### 3.3 Performance of the DEAttentionDTA model in the p38 protein family

The p38 serine/threonine protein kinase family (Cuadrado and Nebreda 2010) is highly conserved in eukaryotes. The signalling pathway involving p38 plays a critical role in cellular stress responses. Virtually all adverse external stimuli and internal changes can activate the p38 signalling pathway in different cell types, making it a major stress-activated signalling pathway. This pathway is implicated in various physiological and pathological processes, such as cell apoptosis, cellular stress, cell cycle regulation, and inflammatory responses. In recent years, several p38 inhibitors have been designed for the experimental manipulation of signal transduction pathways (Gao *et al.* 2019).

**Figure 5.**
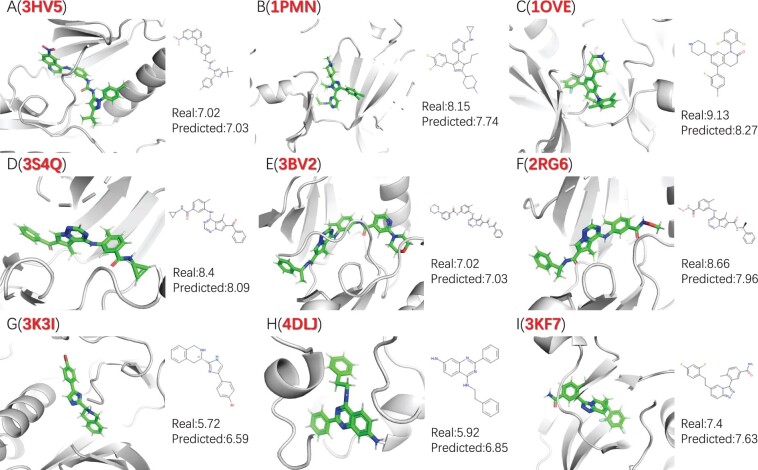
Measurement and prediction of the affinity between p38 proteins and other compounds.

We obtained 154 protein–ligand complexes from the RCSB database belonging to the p38 mitogen-activated protein kinase family to assess the predictive performance of the DEAttentionDTA model. The results indicate an RMSE, MAE, SD, CI, and *R* of 1.079, 0.817, 0.982, 0.796, and 0.833, respectively. Some of the predicted results are presented in Figure 5, and the close resemblance to the actual values highlights the outstanding performance of DEAttentionDTA in predicting the protein–ligand binding affinity.

We compared the prediction results of each model for 154 protein–ligand complexes. The tool with the closest results was considered the top1 prediction tool for that complex. The results showed that out of the 154 complexes, DEAttentionDTA, GraphscoreDTA, DeepDTAF, DLSSAffinity, and Pafnucy obtained the top1 predictions for 60, 33, 21, 22, and 18 complexes, respectively. Compared with the other tools, DEAttentionDTA demonstrated the highest prediction accuracy, whereas GraphscoreDTA also exhibited good predictive performance. DeepDTAF, DLSSAffinity, and Pafnucy fell slightly behind in their prediction results (the complete prediction results, including the pdbid for each of the 154 proteins, are presented in the Supplementary Material).

## 4 Discussion

Protein–ligand affinity prediction plays a crucial role in the early stages of drug development. In this study, we focused solely on predicting the affinity using 1D sequences of proteins and small-molecule ligands. Unlike many mainstream tools based on the 3D structure of proteins, our deep-learning-based tool, DEAttentionDTA, demonstrated superior performance on public datasets. DEAttentionDTA uses three different inputs: amino acid sequences, pocket sequences, and small-molecule ligand sequences. In its v2 version, it utilizes amino acid and small-molecule ligand sequences. This tool incorporates a dynamic word-embedding layer to ensure a unique and distinct word embedding for each amino acid. Subsequently, through the self-attention layer, DEAttentionDTA facilitates better information transfer during long-range interactions within amino acid and small-molecule sequences by utilizing attention blocks to associate amino acid and small-molecule sequences. Additionally, we applied DEAttentionDTA to predict the affinity of 154 protein–ligand pairs related to p38, demonstrating the effectiveness of DEAttentionDTA-based predictions. Our model also had certain limitations: due to the fixed input length of deep-learning networks, some information of sequences and pockets may be lost during truncation. In future work, we hope to use sliding windows to not only truncate the end or beginning of the text but also repeatedly truncate the sequence, and then average the multiple prediction results, to preserve as much information as possible.

In summary, DEAttentionDTA can stand out among the existing tools for protein affinity prediction and provide promising applications for AI-assisted drug development. Our approach will significantly advance the drug screening process by elucidating both the effects of drugs and the underlying mechanisms.

## Supplementary Material

btae319_Supplementary_Data

## Data Availability

The resource codes are available at https://github.com/whatamazing1/DEAttentionDTA.
