# Supplementary material for: DEAttentionDTA: protein–ligand binding affinity prediction based on dynamic embedding and self-attention
Source: Bioinformatics. 2024 Jun 19;40(6):btae319. doi: 10.1093/bioinformatics/btae319 (PMC11193059; doi:10.1093/bioinformatics/btae319)
Supplement: btae319_Supplementary_Data [file btae319_supplementary_data.pdf]

Supplementary Materials

DEAttentionDTA: Protein-ligand binding affinity prediction based on dynamic embedding and self-attention

Xiying Chen, Jinsha Huang, Tianqiao Shen, Houjin Zhang, Li Xu, Min Yang, Xiaoman Xie, Yunjun Yan, Jinyong Yan

Key Lab of Molecular Biophysics of Ministry of Education, College of Life Science and Technology, Huazhong University of Science and Technology, 1037 Luoyu Road, Wuhan 430074, China

***Corresponding author**

Yunjun Yan, yanyunjun@hust.edu.cn

Jinyong Yan, yjiny@126.com

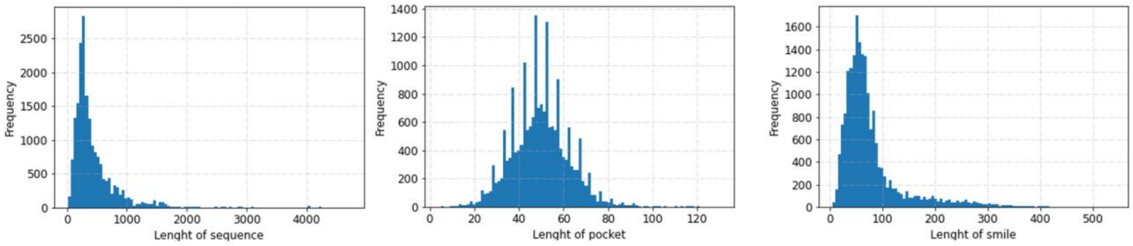

Figure 1. Statistics of the lengths of the whole dataset. (A) Distribution of the lengths of protein sequences. (B) Distribution of the lengths of pocket sequences. (C) Distribution of the lengths of ligand SMILES sequences.

Table 1. Pre-defined dictionaries of ligand sequences

element	label	element	label	element	label	element	label
<MASK>	0	C	1	)	2	(	3
c	4	O	5	]	6	[	7
@	8	1	9	=	10	H	11
N	12	2	13	n	14	3	15
o	16	+	17	-	18	S	19
F	20	p	21	l	22	/	23
4	24	#	25	B	26	\\	27
5	28	r	29	s	30	6	31
I	32	7	33	%	34	8	35
e	36	P	37	9	38	R	39
u	40	0	41	i	42	.	43
A	44	t	45	h	46	V	47
g	48	b	49	Z	50	T	51
M	52						

Table 2. Pre-defined dictionaries of protein sequences

element	label	element	label	element	label	element	label
<MASK>	0	A	1	C	2	D	3
E	4	F	5	G	6	H	7
K	8	I	9	L	10	M	11
N	12	P	13	Q	14	R	15
S	16	T	17	V	18	Y	19
W	20						

Table 3. The p38 mitogen-activated protein kinase family to assess the predictive performance of the DEAttentionDTA model and other competing methods. Display best result in bold with underline.

**id_name**	**real**	**DEAttentionDTA**	**GraphscoreDTA**	**DeepDTAF**	**DLSSA**	**Pafnucy**

1a9u	7.32	6.971498	**7.52966**	8.086921	6.305701	6.73234
1bl6	6.8	**6.607682**	7.814843	6.231521	7.136692	6.18446
1bl7	7.72	**7.285217**	8.759816	6.885516	6.249715	7.073292
1bmk	7.6	6.454725	5.960683	8.991833	**6.902785**	8.783497
1di8	6	**6.15677**	6.299004	7.129356	6.616238	6.933432
1di9	5.3	**5.708275**	4.104697	4.237717	6.535983	3.94102
1kv1	5.94	6.75669	3.863171	**5.6673**	5.289003	5.472462
1kv2	10	7.919181	7.720537	8.668565	8.784587	**10.82046**
1m7q	8.59	8.130103	9.635119	7.182424	**8.140957**	9.769473
1oeb	6.93	5.387761	7.397912	6.11951	**6.533419**	7.437189
1ouk	9.89	8.275887	**9.495214**	8.356316	10.63515	8.321089
1ouy	8.37	**8.235243**	7.248655	8.755732	8.811008	9.298114
1ove	9.13	8.269466	9.726546	7.940102	**8.697639**	8.192947
1pme	9.4	7.297282	8.470856	10.90892	**8.997395**	10.84943
1pmn	8.15	7.741921	**7.772921**	7.628992	8.806781	9.230319
1pmu	6.23	7.078394	**6.439709**	7.238641	7.443207	5.352438
1pmv	6.82	5.98153	8.095143	5.078201	7.821988	**6.328174**
1py5	7.29	**7.215142**	9.256814	8.251674	6.827906	6.948874
1rw8	6.76	**7.401409**	8.052823	5.670779	5.280658	7.675135
1vjy	7.64	**8.259278**	6.180487	9.473962	8.405387	8.734964
1w7h	3	4.423628	**3.368148**	2.017548	1.569841	2.39734
1w82	6.71	7.693471	6.30148	4.63322	7.422106	**7.012077**
1w83	7.19	7.819604	8.28614	**6.776313**	8.30923	6.738494
1w84	4.46	4.910049	5.358209	2.480384	3.022942	**4.741342**
1wax	4.07	5.346528	4.613204	2.898038	**4.609439**	2.993869
1way	3.4	5.01546	**3.114864**	4.511587	5.615052	2.263441
1wbg	3	3.891816	4.057117	3.825675	1.672555	**3.453296**
1wbn	6.46	**6.759431**	5.57716	6.116889	6.156222	5.83506
1wbo	3	3.624368	4.298444	2.40623	**3.588201**	3.671853

1wbs	6.2	7.378333	5.276496	5.603752	5.830706	**5.832744**
1wbt	6.47	**7.218118**	8.510738	7.42363	7.361625	7.548993
1wbv	3.79	6.461118	4.866656	4.36939	**3.586115**	4.052554
1wbw	4.36	5.628853	**3.593828**	2.346637	5.439745	3.202106
1wcc	3.46	**3.638263**	2.962411	2.057801	2.038454	3.733798
1yqj	8.14	**7.87659**	10.56287	8.743485	7.035331	8.610722
1yw2	6.85	**7.026696**	6.572908	6.295333	5.745106	5.661434
1ywr	7.89	**8.069121**	8.40114	6.311722	9.121862	8.366579
1zyj	5.82	6.665515	7.819303	**6.070061**	5.031623	7.441847
1zz2	5.22	6.810808	4.24584	4.616004	3.433302	**4.722635**
1zzl	8.3	7.506425	7.651405	**8.076143**	8.597697	7.812338
2b1p	8.52	7.351741	**9.130197**	7.064133	9.501997	7.177294
2baj	8.4	6.963886	**8.056172**	7.172812	9.067797	9.959214
2bak	7.43	**7.632607**	6.421343	9.190567	6.804121	7.945767
2bal	6.31	7.773968	4.752782	5.828619	7.805066	**6.67677**
2ewa	7.94	**7.334087**	7.18824	7.225052	9.866516	6.900482
2gcd	5.52	8.160166	**5.099867**	6.375538	4.436645	4.641511
2gfs	6.15	**6.254544**	6.846283	5.791757	5.256963	4.395317
2hrp	8.3	6.677073	7.454054	6.517404	**7.749155**	9.32828
2i0h	8.05	**7.866668**	9.075208	6.486425	8.833472	9.369165
2p3g	6.9	7.894429	**7.101911**	6.278507	8.121275	8.302576
2q8y	3.99	6.697411	**3.677145**	2.904151	3.643801	3.5987
2qd9	7.89	**8.239733**	6.173715	9.55667	7.521058	7.077523
2r9s	6.82	8.160581	**7.052222**	6.435968	7.881809	7.623721
2rg5	8.51	7.517415	6.98997	**9.226759**	9.260234	7.397051
2rg6	8.66	7.959926	7.211862	9.454008	7.935788	**8.958107**
2y8o	5.12	**5.528185**	4.01468	6.172715	3.761017	7.048883
2yis	8.77	**8.469152**	8.157345	7.861821	9.954298	9.817971
2yiw	9.1	8.241769	7.618485	8.58823	**9.581236**	9.619314

2zaz	6.4	**6.865247**	7.064054	7.144882	5.348647	5.279202
2zb0	6.62	**6.318622**	3.990371	5.530214	7.176518	7.574202
3bv2	9.36	7.758434	**8.963102**	8.659297	8.799418	10.10273
3bv3	9.34	7.554646	**8.94569**	10.11833	9.870513	10.23385
3bx5	8.46	7.878182	**8.200524**	7.914993	7.925979	10.1444
3c5u	8.19	**8.107711**	7.009883	7.183603	9.450609	7.943135
3ctq	7.4	**7.613945**	7.182758	5.879105	8.508703	5.929547
3d7z	7.92	**7.844655**	9.519099	7.37285	9.768918	9.61191
3d83	8.36	**8.308944**	7.838342	6.05101	10.23896	9.585072
3ds6	9.1	8.478517	7.943742	**8.855264**	10.16813	7.094434
3dt1	8.62	7.454747	**8.301594**	10.61744	9.830533	9.312164
3fc1	8	7.710711	**7.770955**	5.298623	8.754697	8.764873
3fi2	7.6	6.484008	8.690968	6.655608	**8.261846**	6.606667
3fi3	7.92	**7.862431**	8.463533	8.550519	8.722579	6.725714
3gc7	9.22	**8.629704**	9.986158	9.860922	10.1136	8.516831
3gcp	7.82	**7.487172**	7.091612	7.224458	9.98671	7.267833
3gcq	6.47	**6.892559**	5.548831	9.132187	4.35226	7.648143
3gcu	6.78	7.211688	**6.560833**	5.76233	5.744102	6.179647
3gcv	7.13	**7.351929**	6.690152	6.666973	5.867565	7.827003
3gfe	8.49	8.162743	**8.269877**	8.260098	7.290782	9.667152
3gp0	7.44	8.066947	8.386003	6.896545	**7.81759**	8.213277
3hec	4.47	6.984787	4.139614	4.07497	**4.740055**	5.532761
3heg	6.74	7.197182	6.316662	6.16136	7.176451	**6.984169**
3hl7	7.64	8.222344	**8.151809**	6.58889	6.510688	6.835861
3hll	8.24	**7.617133**	7.323481	9.394605	6.72688	7.351086
3hp2	6.17	7.186322	**6.688878**	5.076563	5.353526	5.020832
3hp5	7.15	**7.509189**	8.062925	6.788123	8.854584	5.128449
3hrb	7.68	7.084771	8.745651	6.900872	9.221717	**8.090408**
3hub	8.09	7.764175	7.203942	7.042604	**7.870097**	6.4717

3hv3	6.33	6.948565	**5.869368**	7.036463	7.082152	8.697142
3hv4	6.51	7.36967	7.092071	**6.901662**	4.299149	5.706667
3hv5	7.02	**7.03112**	6.447678	6.240914	4.802419	6.714819
3hv6	5.64	7.537323	**5.327495**	6.184075	6.782788	4.610353
3hv7	7.92	7.077719	**8.4977**	7.262425	8.852323	6.964974
3hvc	6.22	5.769318	7.008781	**5.948117**	6.87089	5.024721
3iph	7.6	8.17489	8.844453	**8.111303**	8.662757	6.511278
3itz	8.85	8.264961	10.02101	8.18412	**8.318657**	7.235285
3iw5	6.08	7.513906	5.059177	**5.367401**	7.937971	8.010723
3iw6	5.48	6.770216	**5.270054**	6.021087	4.596305	5.776512
3iw7	5.64	7.245591	**5.261067**	6.64425	5.117737	6.756668
3iw8	4.87	5.943633	**4.327566**	5.652995	6.934337	3.836516
3k3i	5.72	6.597663	4.978475	6.162847	5.080895	**5.461566**
3k3j	4.9	6.736323	3.139072	5.656067	**5.638618**	3.968306
3kf7	7.4	**7.637546**	8.587309	8.282521	9.64843	9.157227
3l8s	7	7.752726	8.459073	**6.561435**	8.147305	8.195142
3l8x	8	**7.774534**	7.4042	8.366669	6.455945	9.174429
3lhj	9.51	8.145997	8.327521	10.08689	8.787384	**9.087184**
3mpt	6.6	7.803851	5.596088	6.264157	**6.29207**	4.954611
3mvl	7.89	7.521766	6.764111	7.183937	**7.576139**	8.516925
3mvm	8.41	7.825035	7.074631	**8.758848**	9.256964	8.767602
3mw1	9.09	7.983721	8.136448	9.527166	8.540511	**8.695382**
3new	5	7.41478	3.838208	3.049672	6.43697	**4.008175**
3nnu	7.32	8.235759	8.644741	**6.849897**	8.326227	6.22221
3nnv	7.57	**8.029885**	6.973539	8.59168	5.254314	6.139841
3nnw	8.05	**7.941224**	7.008291	7.629349	7.116458	6.964974
3nnx	7.96	**7.815868**	8.902584	6.864291	6.738807	7.077299
3nww	8.15	**8.212514**	7.205359	8.634613	7.937734	7.151475
3ocg	8.52	7.999351	8.063296	8.120191	7.167265	**8.310241**

3p5k	7.12	**7.093057**	8.764209	5.896382	8.333895	6.595013
3p78	6.96	**7.223638**	5.972195	5.950341	6.380724	5.548623
3p79	5.64	7.119971	**5.333781**	4.477978	6.760468	6.166329
3p7a	6.28	7.206279	4.995625	**6.570259**	5.970345	5.234875
3p7b	7.74	**7.411942**	8.812241	6.767279	8.909185	5.783188
3p7c	7.66	7.226309	6.943733	**7.382198**	9.69585	6.542108
3rin	8.89	8.200357	**8.479191**	8.268782	8.161446	8.44641
3roc	8.19	**7.878428**	7.034345	8.962464	7.494531	7.761531
3s3i	7.8	**7.565816**	8.392025	8.674948	8.847645	7.181298
3s4q	8.4	**8.097502**	9.717191	7.507294	9.511889	9.467944
3uvp	7.46	**7.19818**	6.36705	6.692487	8.24095	5.627132
3uvq	7.77	**8.139166**	8.215626	8.726946	9.717795	8.237193
3zya	7	**7.323615**	5.99844	8.343433	7.959695	7.364635
4aa0	7.46	7.823061	6.761381	**7.196279**	6.768174	8.591783
4aa4	8.05	7.576629	6.381638	8.466293	**7.766949**	7.695641
4aa5	7.8	8.157092	9.331096	**8.052528**	9.861026	7.085012
4aac	8.17	**8.151495**	7.101226	7.497222	7.455807	7.735994
4dlj	5.92	6.853354	6.982616	7.44578	**5.241622**	4.916153
4e6c	4.82	**4.54096**	5.32771	3.756387	5.97072	7.248497
4eh2	3.16	5.141098	2.652152	**3.468115**	1.998616	2.513944
4eh3	3.53	5.6832	4.359187	3.098196	**3.803675**	3.851978
4eh4	2.81	4.050868	1.723302	3.472391	3.722582	**2.464798**
4eh5	3.09	4.427534	0.846145	1.988985	4.323822	**2.363765**
4eh6	2.46	4.894515	5.566597	1.268521	**3.354977**	1.25029
4eh7	3.28	4.722518	3.834593	**3.73528**	1.559423	2.515945
4eh8	2.2	4.201972	**2.429691**	3.424099	0.852117	1.445948
4eh9	2.26	6.206269	0.930436	**1.774664**	2.965459	4.296989
4ehv	2.29	5.388974	**2.677198**	1.689973	0.549285	3.309841
4f9y	6.74	**7.224092**	5.733054	5.683646	7.626	5.604475

4l8m	9	**8.51961**	9.697616	8.231461	9.649875	7.431368
4loo	8.15	**8.259275**	9.060895	9.154012	7.723544	10.00055
4lop	8.15	**8.348464**	8.690576	9.756769	8.514134	6.860049
4loq	8.15	**8.239107**	8.70584	9.101764	7.411487	6.952482
4r3c	7	**7.208156**	6.464137	7.698726	6.560818	8.229039
2yix	8.48	**8.07796**	9.845983	7.541631	10.12045	8.980787
2zb1	6.32	7.322641	**5.408396**	5.101832	5.384116	4.726605
4dli	5.62	**6.010823**	6.495491	4.783925	4.520552	6.368703
4f9w	6.94	6.657513	8.309151	**7.188272**	5.81336	7.995916
total		60	33	21	20	18
